# Paradigm shifts: exploring AI's influence on qualitative inquiry and analysis

**DOI:** 10.3389/frma.2024.1331589

**Published:** 2024-12-05

**Authors:** Ryan Thomas Williams

**Affiliations:** Teesside University International Business School, TU Online, Teesside University, Middlesbrough, United Kingdom

**Keywords:** artificial intelligence, qualitative data analysis, language processing, paradigms, interpretivism

## Abstract

Technology has mostly been embraced in qualitative research as it has not directly conflicted with qualitative methods' paradigmatic underpinnings. However, Artificial Intelligence (AI), and in particular the process of automating the analysis of qualitative research, has the potential to be in conflict with the assumptions of interpretivism. The short article aims to explore how AI technologies, such as Natural Language Processing (NLP), have started to be used to analyze qualitative data. While this can speed up the analysis process, it has also sparked debates within the interpretive paradigm about the validity and ethics of these methods. I argue that research underpinned by the human researcher for contextual understanding and final interpretation should mostly remain with the researcher. AI might overlook the subtleties of human communication. This is because automated programmes with clear rules and formulae do not work well-under interpretivism's assumptions. Nevertheless, AI may be embraced in qualitative research in a partial automation process that enables researchers to conduct rigorous, rapid studies that more easily incorporate the many benefits of qualitative research. It is possible that AI and other technological advancements may lead to new research paradigms that better underpin the contemporary digital researcher. For example, we might see the rise of a “computational” paradigm. While AI promises to enhance efficiency and rigor in data analysis, concerns remain about its alignment with interpretivism.

## Introduction

The trustworthiness and credibility of data often characterize research that is underpinned by qualitative research. In other words, qualitative research often requires the researcher to interpret data, and these interpretations are shaped by the researcher's experiences, backgrounds, and biases (Cohen et al., 2018). Depending on the researcher's paradigmatic position and assumptions, this is usually viewed as a justification for or against conducting qualitative research.

An interview, for instance, may involve what has been termed commodification of the skills of “building a rapport” where the researcher may engage in the unethical affair of “faking friendship” to obtain knowledge (Duncombe and Jessop, 2002). Generally, researchers uphold a monopoly of interpretation over the subjects' statements and can interpret and report what the subjects really meant due to this rapport-building process.

Whilst technology has influenced the interview process through voice recordings, online interviews, and, more recently, speech-to-text technology, unlike in quantitative research, when conducting a qualitative study, the researcher is still considered the main instrument for data collection, data analysis and data interpretation (Paisley and Reeves, 2001). Lincoln and Guba (1985, p. 236) argue that “the instrument of choice in naturalistic inquiry [qualitative research] is the human”. As such, in qualitative research, researchers bring their inherent (researcher) biases, which are acknowledged and identified in the social sciences. In other words, subjectivity is present in qualitative research, and the contextual understanding of the phenomena is usually through reflexivity and an understanding that the social world is complex.

Qualitative analysis can produce a rich understanding of a phenomenon but requires an essential data annotation process known as coding. Thematic analysis (TA), for instance, is one of the most popular qualitative data analytic techniques in psychology and the social and health sciences (Braun and Clarke, 2006), yet it is critiqued for being time-consuming, particularly for large datasets (Braun and Clarke, 2019). The natural evolution of using technology for coding, such as NVivo and Computer-assisted qualitative data analysis software (CAQDAS), has benefitted academics as it allows researchers to organize and manage sources in one place, importing and coding different types of data, such as PDFs, Word documents, web pages, audio, and video (Kelle and Bird, 1995).

However, researchers are now using Natural Language Processing (NLP)- in other words, a component of Artificial Intelligence (AI)- algorithms beyond identifying meta-inferences in quantitative research and now into the realms of qualitative research to identify key themes and underlining meanings of interview data (Chang et al., 2021).

Nevertheless, the introduction of AI in qualitative research to automate the coding of large data sets to broaden the feasibility of large-scale qualitative research is perhaps in conflict with some of the paradigmatic underpinnings of interpretivism. Interpretivists argue that qualitative analysis should be nuanced, contextual, and human-centered, and there is concern that AI might overlook the subtleties of human communication (Lennon et al., 2021). In interpretivism, a single phenomenon may have multiple interpretations rather than a truth that a measurement process can determine. Furthermore, interpretivists embrace the notion that different researchers may come to different conclusions when coding and analyzing the same data (Hammersley, 2012).

Automation involves designing software or hardware capable of automatically doing things without any human intervention (Shekhar, 2019). Therefore, it must be noted that the process of automation does involve, at the very least, human programming and coding. Samuel (1962) argues that coding determines how much you will be able to make a system simulate a human. In other words, automation in terms of qualitative data analysis is influenced by the data, subtleties, and biases that are fed into it through algorithmic logic, which is not dissimilar to the subjectivities acknowledged with humans analyzing qualitative data. The author argues that automated qualitative software does involve some human decision-making.

In this paper, I explore AI technologies, such as traditional NLP tools and how they have started to be used to analyze qualitative data, such as interviews and open-ended survey responses. While this can speed up the analysis process, it has also sparked debates within the interpretive paradigm about the validity and ethics of these methods. The paper aims to answer how AI impacts the trustworthiness, credibility, and ethical considerations of qualitative research methodologies, specifically in the context of data analysis.

In ethics, for instance, participants are typically provided a date by which they should inform the researcher of their desire to withdraw. However, an ethical issue arises when AI has already been used to analyze the data (Williams, 2024). The underlying algorithms of the technology will have already learned from the inputted data, potentially making withdrawal not possible at this stage. In other words, true deletion of data may not be possible with AI. An additional ethical concern is that the General Data Protection Regulation (GDPR) and the UK Data Protection Act 2018 (DPA) state that individuals have the “right to be informed” about how their data is processed; automating the analysis processing using AI, means this may not be possible as overall algorithmic transparency is low (Meyer von Wolff et al., 2020). Researchers can confirm the algorithmic construction if known, but there are few details on how it is implemented and its knowledge bases.

Nonetheless, there are challenges beyond ethical considerations, including questions about the nature of qualitative research and its purpose.

## Paradigm wars and technology

Educational research has several competing views of the social sciences, often referred to as paradigms. Hammersley (2012, p. 13) portrays paradigms as “not simply methodologies; they are ways of looking at the world, different assumptions about what the world is like and how we can understand or know about it”. In other words, a paradigm is a way of pursuing knowledge through a shared set of beliefs and principles (Hammersley, 2012; Kuhn, 1962). Despite the validity and importance of qualitative and quantitative research approaches, the different positions and assumptions have punctuated research capacity and academic development. This philosophical discourse led to the rise of the term “paradigm wars” with some academic commentators using war-like terminology to describe positions, such as “enemies,” “opposing armies,” and “treat former enemies with suspicion” (see Bryman, 2006; Griffiths and Norman, 2013; Polio, 2012; Williams, 2020).

The rise of mixed methods research demonstrated an interest in embracing varied approaches to collecting and analyzing data in the social sciences. For instance, Polio (2012, p. 294) argued that as future researchers begin to talk about the purpose of the research rather than the paradigm, it opens the door for mixing orientations and is a way to move “beyond the paradigm wars”. Mixed methods have been viewed as a transformative paradigm. Nevertheless, in this paper, I argue that technology, in particular, automation of qualitative data analysis through NLP, has influenced how we view these debates. Qualitative researchers should reconsider the concept of paradigms and reclaim the paradigmatic stance that avoids algorithms with rigid rules and formulae and embraces the subjectivity involved in qualitative inquiry.

While qualitative and quantitative methods may be used appropriately with any research paradigm, interpretive and critical theory paradigms are central to qualitative research (Guba and Lincoln, 1994).

Tools such as CAQDAS have been commonly used to assist researchers with managing, organizing, and analyzing qualitative data (Kuntsche et al., 2022; Lewins and Silver, 2014). These software programs began as mechanisms to better organize and code data and now include analytic tools such as word frequencies, word clustering, sentiment analysis and thematic analysis. These features assisted researchers in constructing themes from large datasets but still required manual coding of data within the software package. For example, a researcher interviewing teachers across different phases about their attitudes toward education technology could benefit from software such as CAQDAS as a way to identify and organize patterns such as “funding” and “professional development”.

Nevertheless, the meaning behind these terms still lies with the researcher. The process of coding itself remains a time-consuming, labor-intensive, and human process. AI has been suggested as a way to reduce burdens such as the time and cost of annotating qualitative data through automation such as NLP (Abid et al., 2020; Lennon et al., 2021).

## Problems with natural language processing

Natural language processing (NLP) has been used to code qualitative data in exploratory research (Abid et al., 2020; Lennon et al., 2021). In simple terms, the goal of NLP is for computers to not only read documents but to understand the text and the contextual nuances of the language within them. This is significantly different from the technology previously used in qualitative research, such as Nvivo and CAQDAS. In other words, the “understanding the context” part has always been a human process.

Interestingly, Guetterman et al. (2018) explored augmenting qualitative text analysis with language processing software and found that an initial modeling technique could generate topic categories from which the researcher identified overall themes similar to traditional methods (human analysis). The study involved text messaging qualitative survey questions to participants, which is somewhat different from the depth of data obtained from an interview; however, AI identified similar key themes as a human, but in a shorter time. However, Guetterman et al. (2018) recommended that NLP be used as a step one to identify major themes or produce mind-maps, as currently, the technology cannot identify nuances in the text. Traditional qualitative text analysis added important details and context that added credibility and trustworthiness to the data. This is relevant when considering how AI could be embraced in qualitative research rather than dismissed because of the theoretical frameworks.

Another study by Lowe and Berry (2020) found that language analysis produced similar outcomes to traditional qualitative methods when analyzing topics on Twitter (now X). More specifically, the technology produced a hierarchy of data, similar to the hierarchical structure of code-subtheme-theme in thematic analysis. AI, such as NLP, may be useful for evaluating short text, but when data includes detailed and rich data, it cannot fully identify nuances within the data (Lowe and Berry, 2020; Guetterman et al., 2018). In this sense, AI tools such as NLP may be viewed as just another tool such as NVivo and CAQDAS that qualitative researchers can draw upon.

Researchers should be aware of artificial hallucinations when evaluating AI insights. Hallucinations are responses generated by an AI, such as a language model which contains false or misleading information presented as fact (Ji et al., 2022). For example, a hallucinating generative chatbot might falsely state that interpretive studies were positivist when asked. Alternative terms such as *faithfulness* and *factuality* have been proposed to assess the accuracy and adherence more accurately to external knowledge sources of AI-generated content (Dong et al., 2020).

Emsley (2023) cautions educators and researchers about the falsifications that can be generated using AI. In a study investigating the authenticity and accuracy of references in case studies generated by Chat Generative Pre-trained Transformer (ChatGPT), Emsley (2023) found that of 115 references that were generated, 47% were fabricated, 46% were authentic but inaccurate, and only 7% were authentic and accurate, and this raises questions about the reliability of using AI to automate the data analysis process (Williams, 2023).

Regardless, this paper is not a critique of the technology itself; as language analysis develops, features will undoubtedly be associated with it that involve a deeper understanding of the data, enhancing reliability and validity. For example, Large Language Models (LLMs) represent a significant advancement in the broader field of NLP, and may be able to capture the nuances and awareness required for qualitative research in the future (Bano et al., 2023). Bano et al. (2023) argue that LLMs such as Generative Pre-trained Transformer (GPT) can capture relationships between words, phrases and sentences in a contextually meaningful way, closely drawing to the sophistication of human interpretations, for instance. Additionally, researchers can fine-tune LLMs by asking them to explain their reasoning through annotating outputs, references to data patterns, and transparency in coding decisions. This is similar to a human researcher providing a rationale during thematic analysis, making LLMs a versatile and potentially useful tool for qualitative analysis (Alawida et al., 2023). However, the feasibility of relying solely on such explanations must be evaluated, as AI may fabricate plausible yet unverifiable interpretations (Bano et al., 2023). In other words, LLMs will still have to grapple with several challenges, including ethical concerns related to bias, the absence of true reasoning capabilities, and their lack of emotional understanding.

This paper examines phenomena in terms of what it means for interpretivism and a researcher's interpretation, belief system, ways of thinking, cultural preference, and bias. By using AI to analyze qualitative data, a researcher may neglect the theoretical perspective that underlies it. However, the most obvious advantage of using AI to produce large amounts of data is efficiency and speed. This could be particularly useful to studies that involve time-sensitive research questions like those related to COVID-19 behaviors. Additionally, researchers may benefit from using NLP to analyze data in a different language. Such an approach would not require the researcher to be fluent in that language, yet would allow them to analyze the data (Abid et al., 2020).

Automating interview data may encourage researchers to make claims of reproducibility as AI algorithms underpin the data can make the analysis process more transparent and reproducible, given that the same data input and algorithm should produce the same results. It is important to note that different AI systems may produce different results. Reliability is concerned with how consistently a method measures something. The measurement is considered reliable if the same result can be consistently achieved by using the same methods under the same circumstances. Nevertheless, qualitative research does not conform to the same reliability and validity rules as quantitative. Rather, data quality in qualitative research is determined by the trustworthiness of data (Lincoln and Guba, 1985). Trustworthiness is achieved by credibility, authenticity, transferability, dependability, and confirmability. In other words, Interpretivists prefer qualitative research methods and are prepared to sacrifice reliability and representativeness to gain deeper insight, which should provide higher validity. Therefore, the notion of reproducibility being a particular strength of AI is questionable.

The rise of AI has the potential to disrupt the qualitative research process. With the development of natural language processing and other AI technologies, it is becoming increasingly possible for machines to analyze human language (Abid et al., 2020). However, qualitative research is underpinned based on the assumption that reality is subjective, multiple, and socially constructed. That is to say, we can only understand someone's reality through their experience of that reality, which may be different from another person's shaped by the individual's historical or social perspective (Cohen et al., 2018). Thus, automated programs that consist of a clear set of rules and formulae consistently followed each time do not work well-under interpretivism's assumptions. Researchers who use a general artificial intelligence agent only cover the rules-based epistemological spectrum of positivism. In other words, there is little scope for interpretive studies to be analyzed solely by mathematical algorithms.

## Discussion of potential solutions: emerging paradigms

It is possible that AI and other technological advancements may lead to new research paradigms that better align with the increasingly contemporary digital researcher. For example, we might see the rise of a “computational” paradigm, which can be described as a paradigm covering both quantitative and qualitative research elements, relying on AI algorithms to analyze complex, large-scale datasets while still recognizing the importance of human context and interpretation. This paradigm shift may be considered a fourth paradigm underpinned by data science (Goodfellow et al., 2016; Jurafsky and Martin, 2019). This is not to say that AI algorithms alone may not be capable of the different subtleties of qualitative research. Turing (1950) and McCarthy et al. (2006) have long asked questions about whether computers can really think and mimic humans, leading to the development of Fuzzy Logic (FL) theories, for instance. FL is a method of reasoning based on vague and imprecise information and resembles human reasoning (Klement and Slany, 1993).

In terms of methodological underpinnings, data extends from small-scale interviews into large-scale digital data, challenging traditional clear-cut distinctions between objectivity and subjectivity. While computational methods are often seen as objective due to their rules-based nature, their application in qualitative research requires an acknowledgment of the subjectivity inherent in how algorithms are designed, data is interpreted, and results are contextualized. In qualitative research, this means acknowledging that part of the knowledge construction process can be algorithmically driven, supplementing human interpretation and analysis. In other words, researchers may acknowledge how data-driven insights can complement and enrich their understanding of subjective experiences and social constructs.

Furthermore, using computational methods such as AI has implications in terms of reflexivity, extending to include the researcher's relationship with technology.

One of the key elements of a computational paradigm is the ability to handle and analyze “big data”- large datasets that are too complex to be dealt with using traditional data-processing methods. With AI and machine learning algorithms, researchers could perform language analyses on these datasets, identifying patterns and key themes in the text and interpreting these results in the context of social theory. This concept is interdisciplinary as it would draw on methods and theories from computer science, statistics, social sciences, and other fields. However, there is an assumption that the researcher ultimately influences the process, and various tasks performed during the research, such as designing the interpretative repertoire and inputting training data, are influenced by the researcher's ideas, beliefs, attitudes and biases.

However, a computational paradigm must grapple with new ethical and privacy issues. For instance, big data analyses often involve personal data, which raises questions about consent and data protection. Who owns the data once you use an automated processor to analyze the data? Does the computer use this input to learn for future outputs? What happens when the participants withdraw from the study, but the data has already been entered into the AI NLP? What happens to the data afterwards? Hasal et al. (2021) states that if a chatbot can access the personal data of a user, the chatbot must have the GDPR mandates and regulations in place. Universities and educational institutions must establish clear and robust data collection, storage, and usage guidelines, strictly aligning with legislations such as the General Data Protection Regulation (GDPR) in the EU and the Children's Online Privacy Protection Act (COPPA) in the US. However, this is far more complex in practice.

Interestingly, current UK data privacy regulations allow individuals to request that their data be deleted from an organization after a certain period, yet the underlying algorithms of the technology will have already learned from the inputted data; thus, true deletion of data may not be possible. Furthermore, another concern is that GDPR and the DPA provide individuals with the “right to be informed” about how their data is processed; however, some scholars have argued that this will “never” be revealed by companies and that algorithmic transparency is low, which challenges data protection legislation (Meyer von Wolff et al., 2020).

Similarly, using AI in research raises new ethical questions, such as the potential biases in AI algorithms (Williams and Ingleby, 2024). Like all AI systems, chatbots learn from large amounts of data gathered from the internet, which unavoidably represents societal biases. If the data used to train these models contains biased attitudes, the AI system will likely assimilate and reproduce these biases, even unintentionally (Bolukbasi et al., 2016). This could manifest as gender, racial, or other biases, significantly impacting the data analysis process experience and worldview when surfaced in a research context. For instance, Anis and French (2023) found that an AI system used by Amazon to identify key skills of an applicant was found to discriminate against women by ignoring phrases such as “women's chess club captain” as evidence of leadership. The system found leadership phrases and skills in male applicants more accurately, largely because of the training data (Stahl et al., 2023).

While a computational paradigm could offer innovative research approaches, it could also overcome some limitations of traditional paradigms and facilitate a more holistic and comprehensive understanding of complex phenomena, such as AI. However, it also presents new challenges and ethical issues that must be addressed.

I argue that research underpinned by a computational method and paradigm will likely necessitate a fundamental shift toward a more reflexive research process. The intricate nature of machine learning algorithms and the burgeoning ethical landscape of AI necessitate not only heightened awareness of researchers' own potential biases in data selection and its subsequent influence on algorithmic outputs but also the mitigation of these biases alongside those inherent within the AI itself (Hirblinger et al., 2023).

Interestingly, the solution may not necessarily be as complex as first imagined. Post-positivists, for instance, recognize that how knowledge is obtained is inherently imperfect and biased (Popper, 1968, 1980). This recognition has become increasingly relevant with the rise of technology. For instance, using AI in social research has brought to light the biases inherent in these algorithms. As a result, post-positivists might argue for a more critical and diverse approach to using technology in research. In other words, biases and shortfalls of using AI in qualitative research can be acknowledged and then mitigated in a reflexive way.

## Conclusion

Technology in qualitative research has been mostly embraced, as with CAQDAS and NVivo for coding purposes. Technology has benefitted academics by allowing researchers to organize and manage sources in one place, importing and coding different types of data, such as PDFs, Word documents, web pages, audio, and video. However, researchers have begun using AI to analyze qualitative data, such as interviews (Chang et al., 2021). Interpretivists argue that qualitative analysis should be contextual and human-centered, and there is concern that AI might overlook the subtleties of human communication (Lennon et al., 2021). Thus, automation may be in conflict with interpretivism.

Whilst AI may be useful for quantitative research, as it can handle large-scale data sifting, pattern recognition, and preliminary coding. Research that is underpinned by the human researcher for contextual understanding and final interpretation should mostly remain with the researcher. This is because automated programs that consist of a clear set of rules and formulae do not work well-under interpretivism's assumptions. In other words, there is little scope for interpretivist studies to be analyzed only by mathematical algorithms. Automation may remove the researcher's experiences, backgrounds, and biases (Cohen et al., 2018). However, automation presents a new set of challenges that can similarly contain bias, be incorrectly coded, or be prone to AI hallucinations.

I argue that researchers should balance the benefits of technological advancements with the core philosophies of qualitative inquiry. Nevertheless, AI may be embraced in qualitative research in a partial/hybrid automation process that enables researchers to conduct rigorous, rapid studies that more easily incorporate the many benefits of qualitative research (Lowe and Berry, 2020; Guetterman et al., 2018). In other words, AI could be used for data preprocessing and preliminary analysis, followed by in-depth qualitative analysis conducted by human researchers. There is particular relevance of the AI argument in qualitative research to Richards and Richards (1994, p. 445) assertion that computers offer no instant solutions to the problems faced by qualitative researchers. Thus, researchers should continue to engage in reflexivity on the impact of automation on research outcomes.

It is possible that AI and other technological advancements may lead to new research paradigms that align with the contemporary qualitative researcher. For example, we might see the rise of a “computational” paradigm. This short think piece is not a deep exploration into rising paradigms; thus, future research should fully explore AI's involvement in data interpretation and how this might lead to new research paradigms or alter existing ones. However, firstly, future research is needed to determine the extent to which automation may be applied to qualitative data and the extent to which the algorithms used to augment coding may also be used to augment category development. Future research should also explore the development of AI tools specifically designed for qualitative research, focusing on how they can align with the interpretivist paradigm.

In summary, whilst AI can speed up the process of analyzing vast amounts of qualitative data and has been seen to discover patterns and themes in data (Guetterman et al., 2018), it is in conflict with the notion of multiple interpretations and interpretivism.

## Data Availability

The original contributions presented in the study are included in the article/supplementary material, further inquiries can be directed to the corresponding author.
